# The Quality Prediction of Olive and Sunflower Oils Using NIR Spectroscopy and Chemometrics: A Sustainable Approach

**DOI:** 10.3390/foods14132152

**Published:** 2025-06-20

**Authors:** Taha Mehany, José M. González-Sáiz, Consuelo Pizarro

**Affiliations:** Department of Chemistry, University of La Rioja, 26006 Logroño, Spain; taha.abdellatif@unirioja.es (T.M.); josemaria.gonzalez@unirioja.es (J.M.G.-S.)

**Keywords:** antioxidants, deep-frying, edible oils, lipid oxidation, machine learning, multivariate calibration, regression models, SELECT algorithm, organoleptic markers, vibrational spectroscopy

## Abstract

This study presents a novel approach combining near-infrared (NIR) spectroscopy with multivariate calibration to develop simplified yet robust regression models for evaluating the quality of various edible oils. Using a reduced number of NIR wavelengths selected via the stepwise decorrelation method (SELECT) and ordinary least squares (OLS) regression, the models quantify pigments (carotenoids and chlorophyll), antioxidant activity, and key sensory attributes (rancid, fruity green, fruity ripe, bitter, and pungent) in nine extra virgin olive oil (EVOO) varieties. The dataset also includes low-quality olive oils (e.g., refined and pomace oils, supplemented or not with hydroxytyrosol) and sunflower oils, both before and after deep-frying. SELECT improves model performance by identifying key wavelengths—up to 30 out of 700—and achieves high correlation coefficients (R = 0.86–0.96) with low standard errors. The number of latent variables ranges from 26 to 30, demonstrating adaptability to different oil properties. The best models yield low leave-one-out (LOO) prediction errors, confirming their accuracy (e.g., 1.36 mg/kg for carotenoids and 0.88 for rancidity). These results demonstrate that SELECT–OLS regression combined with NIR spectroscopy provides a fast, cost-effective, and reliable method for assessing oil quality under diverse processing conditions, including deep-frying, making it highly suitable for quality control in the edible oils industry.

## 1. Introduction

The Mediterranean diet plays a crucial role in preventing diseases like cancer and cardiovascular conditions, with extra virgin olive oil (EVOO) being a key component due to its rich phenolic compounds and bioactive substances that provide antimicrobial, antioxidant, anti-inflammatory, antitumor, and antidiabetic benefits [1,2]. EVOO helps to reduce inflammation, lower blood glucose and LDL oxidation, and improve metabolic health [3]. It consists of the following two main fractions: the saponifiable portion (98%), primarily made up of monounsaturated fatty acids (MUFAs) like oleic acid (55–83%), and the unsaponifiable portion (1–2%), containing bioactive compounds such as polyphenols, tocopherols, and carotenoids, which are largely lost in refined olive oils [4]. The health benefits of EVOO are mainly attributed to its polyphenols, especially hydroxytyrosol (HTyr), tyrosol (Tyr), and secoiridoids, known for their potent antioxidant, anticancer, and anti-inflammatory properties, as well as their contributions to EVOO’s distinctive taste and nutritional value [5]. In recent years, global olive oil imports, exports, and consumption have steadily increased, driven by the rising demand for high-quality, health-conscious oils [6].

European legislation classifies virgin olive oil (VOO) based on chemical indicators and sensory attributes, evaluated through a Panel Test (PT). Extra virgin olive oil (EVOO), the highest quality category, requires no sensory defects and a positive fruity attribute. However, this classification does not account for the nutraceutical benefits and sensory richness of EVOO, which are primarily attributed to antioxidants such as tocopherols and polyphenols, characteristic of *Olea europaea* L. The International Olive Council (IOC) sets sensory standards that focus on positive attributes like pungency, bitterness, and fruity notes, while ensuring the absence of defects like rancidity and winey flavors, reflecting olive biodiversity and producer expertise and reinforcing EVOO’s premium quality [7,8]. Sensory analysis by trained panelists, though essential for quality evaluation, is time-consuming. It requires extensive training to assess attributes like fruity, bitter, and pungent and defects such as rancidity and winey flavors. The process, which ensures accuracy and consistency, can take hours per sample and requires a controlled environment to avoid external influences [9]. Given the time and cost of maintaining a professional sensory panel, alternative methods like NIR spectroscopy could offer a practical solution for rapidly screening sensory characteristics and other quality indices, making it a valuable tool for assessing olive oil quality more efficiently [10].

Olive oil’s distinctive color is determined by its pigment composition, which evolves over time and is influenced by various factors, including the olive cultivar, climate, fruit ripeness at harvest, storage conditions, and production process. These pigments, exclusively synthesized by plants and acquired through the human diet, are classified into the following two main groups: carotenoids and chlorophyll derivatives. Beyond their role in coloration, these bioactive compounds possess antioxidant and nutraceutical properties, making their prediction and quantification essential for evaluating oil quality and authenticity [11]. Mínguez-Mosquera et al. [12] introduced an approach that defines two indices, K_670_ and K_470_, based on absorbance measurements at 670 nm and 470 nm, respectively. However, this method has not been validated and involves the use of toxic reagents and chemicals. Despite these limitations, it has been widely applied in various studies due to its simplicity and speed, aiding in the estimation of the total concentrations of chlorophyll derivatives and carotenoids in the chemical–physical characterization of olive oils [13,14].

Furthermore, in accordance with current European olive oil legislation, which is based on the latest implemented regulations and official methods from the Olive Oil Council, conventional olive oil analysis remains time-consuming and reliant on chemical reagents. This limits the efficiency of both official and routine control laboratories, with delays in results potentially affecting EVOO labeling compliance. Developing a single multiparametric method for rapid screening and detecting atypical oils would improve food fraud enforcement and optimize olive oil quality control [15,16,17].

A recent study by Borello and Domenici [14] analyzed the total carotenoid and chlorophyll derivative contents in VOOs and EVOOs from Italy using two spectroscopic methods. The first method calculated K_670_ and K_470_ indices based on absorbance at 670 nm and 470 nm. The second method applied mathematical deconvolution to determine the concentrations of β-carotene, lutein, pheophytin A, and pheophytin B. By comparing both approaches, the study evaluated the effectiveness of spectroscopic techniques for rapid pigment analysis in olive oil. Additionally, NIR spectroscopy could serve as a valuable tool for making real-time decisions during processing, allowing for adjustments to technological parameters like temperature and time during the malaxation step, which directly influence the minor composition of the final VOO [18]. In addition, a chemometric analysis of NIR spectral data (12,500–4000 cm^−1^), along with analytical and sensory parameters, was conducted on 97 monovarietal Cornicabra VOOs produced in the PDO “Montes de Toledo” region. This approach led to the development of calibration and validation models capable of predicting the minor composition of VOO, such as phenolic and volatile compounds, as well as organoleptic attributes, providing a comprehensive assessment of product quality. Strong correlations were found for the degree of unsaturation (fatty acid profile, r = 0.84–0.96), hydroxytyrosol derivatives (r = 0.86–0.88), C6 alcohols (r = 0.69–0.80), and sensory characteristics like ‘fruity’ (r = 0.87) and ‘bitter’ (r = 0.85). The findings confirmed that near-infrared spectroscopy is an effective and rapid tool for evaluating VOO’s minor components and sensory attributes, enabling real-time quality control during production [9].

Recent advances in edible oil authentication increasingly rely on the combination of chemical profiling and chemometric analysis. For instance, Rigakou et al. [19] investigated 223 olive oil samples from different cultivars grown in the Ionian Islands using headspace solid-phase microextraction coupled with gas chromatography/mass spectrometry (HS-SPME-GC/MS). Chemometric techniques such as multivariate analysis of variance and linear discriminant analysis were applied to the volatile compound data—alcohols, aldehydes, esters, and terpenoids, among others—revealing significant differences between cultivars. These analyses enabled the identification of cultivar-specific aroma fingerprints and the construction of robust statistical models for olive oil authentication. This study exemplifies how chemometrics can effectively interpret complex chemical data to support the accurate and rapid classification of olive oils based on their chemical signatures [19].

In addition, a few studies have examined the feasibility of low-cost instruments such as infra-red spectroscopy for assessing the sensorial properties of various EVOO varieties, refined olive oil, and VOO, which would make rapid screening more accessible to EVOO and olive oil producers. NIR spectroscopy is generally considered as a low-cost analytical technique compared to traditional methods such as gas chromatography (GC) and high-performance liquid chromatography (HPLC). In addition to lower instrument costs, NIR offers operational advantages such as minimal sample preparation and no need for reagents or solvents, reducing both time and running costs. While exact prices may vary by manufacturer and market conditions, NIR remains more accessible and environmentally friendly. However, its analytical performance relies heavily on the development of robust calibration models, and its sensitivity and specificity are typically lower than those of chromatographic techniques [20,21,22].

Moreover, to our knowledge, there is no comprehensive report on the application of NIR combined with multivariate analysis to predict pigments, antioxidant activity, and sensorial markers in EVOOs, VOOs, and sunflower oils.

The SELECT approach was applied in the current research to NIR spectra to extract a minimal yet highly informative set of significant predictors. This approach was chosen for its advantages in spectroscopic applications, in contrast to traditional variable selection methods that primarily identify highly correlated variables. Additionally, SELECT has been designed for regression and classification tasks, particularly in spectroscopy and chemometrics, and it selects variables iteratively, ensuring that each new addition provides complementary information while reducing redundancy. By focusing on minimally correlated variables, SELECT enhances model interpretability and performance. Its stepwise decorrelation procedure further minimizes redundancy among selected predictors, improving the calibration model’s efficiency and accuracy [23,24,25]. SELECT, as a variable selection technique, is applicable to both classification [26] and regression [24]. Furthermore, ordinary least squares (OLS) is a widely used regression method based on few selected variables in multivariate calibration [27,28]. Furthermore, the method offers key advantages in multivariate calibration and predictive modeling, operating under assumptions of linearity, independence, and the normality of residuals, which contribute to the reliability and robustness of models [22,29]. Additionally, while PLS is often more effective than OLS for small datasets with high multicollinearity, OLS was appropriate for our current study due to the sufficiently large sample size and the prior dimensionality reduction achieved through SELECT [30]. Moreover, OLS offers advantages in terms of simplicity, clarity, and ease of interpretation, making it particularly suitable for straightforward predictive modeling [31]. Thus, the SELECT + OLS approach offers key advantages for modeling high-dimensional NIR data. It ensures fast, interpretable, and reproducible variable selection, reduces overfitting, and improves model robustness. This method is computationally efficient, easy to implement, and well-suited for real-time quality assessment in food and oil analysis.

The current study aims to develop a robust predictive model that combines spectral analysis optimization with SELECT–OLS regression to quantify key characteristics i.e., pigments (carotenoids and chlorophyll), radical scavenging activity, and sensory markers (rancid, fruity green, fruity ripe, bitter, and pungent), in several edible oils. The model is applied to nine EVOO varieties (including original, hydroxytyrosol-supplemented, and blended), several lower-quality olive oils (e.g., mixtures of refined olive oil with EVOO or virgin olive oil, and pomace olive oil), and different sunflower oils under deep-frying conditions. By evaluating oil composition across eight experimental frying scenarios (3–6 h at 170–210 °C), this approach aims to streamline quality certification and enhance industry standards through the rapid, non-destructive, and sustainable screening of the thermal stability and organoleptic properties of vegetable oils.

## 2. Materials and Methods

### 2.1. Materials and Samples

Cyclohexane (≥99.8%) was purchased from VWR (VWR International, Paris, France). DPPH (2,2-diphenyl-1-picrylhydrazyl) with 99.13% purity was purchased from Sigma-Aldrich (Saint Louis, MO, USA). Methanol with ≥99.9% purity was provided by Fisher Scientific Ltd. (Loughborough, UK). Olive fruit dry extract (20% hydroxytyrosol) was obtained from Natac BioTech (Natac, HQ-Europe, Alcorcon, Madrid, Spain). This study investigated various vegetable oils purchased from local Spanish suppliers, including 9 types of EVOO from different varieties (these varieties represent some of the most prominent and widely consumed Spanish cultivars, ensuring relevance and diversity for the objectives of this study, and include Picual, Cornicabra, Empeltre, Arbequina, Hojiblanca, Manzanilla Cacereña, Royuela/Arróniz, Koroneiki, and Arbosana), 1 EVOO mixed with ROO (called olive oil 1°), 1 pomace olive oil (refined pomace olive oil mixed with EVOO, also known as Orujo oil in Spain), and 1 VOO mixed with ROO (called olive oil 0.4°), as well as refined sunflower oil and refined high-oleic sunflower oil. A total of 11 samples from each olive oil category were analyzed, including controls (non-fried oils and supplemented oils) and 8 samples subjected to deep-frying (D-F) experiments under different conditions (time, temperature, and polyphenol addition). Additionally, 5 samples from each sunflower oil category were also studied. A total of 142 samples were examined, covering all oil types, control samples, and those exposed to deep-frying conditions. For each sample, conventional reference analyses were conducted using UV–vis spectrophotometry to measure parameters such as carotenoid and chlorophyll content and DPPH scavenging activity, while sensory evaluations were performed by a panel. All tests were conducted in triplicate, and 426 spectra were recorded using NIR spectroscopy.

### 2.2. Exogenous Polyphenol Supplementation of Olive Oil

Olive oils were enriched with a hydroxytyrosol-rich olive fruit extract (OFE) to enhance their properties. The process involved fortifying the original oils (EVOO, VOO, or refined olive oils, designated as Control 1) to create a polyphenol-rich supplemented oil, which was then blended with the original oil to form Control 2. According to Mehany et al. [8], OFE was dissolved in water and combined with olive oil, followed by stirring for one hour at room temperature using an IKA-WERKE magnetic stirrer (Staufen, Germany). The mixture was subsequently centrifuged at 9961× *g* for 20 min using a Sorvall RC-6 Plus centrifuge (Osterode, Germany), and the supplemented oil was stored in an amber container at 7 ± 2 °C for further analysis.

### 2.3. Deep-Frying Process

Various olive oil and sunflower oil categories were heated using a 0.5 L volume flask with a Soxhlet heating instrument (SELECTA, Barcelona, Spain). For each deep-frying (D-F) experiment, 0.4 L of oil was placed in the fryer and heated continuously at 170 ± 10 °C for 3 and 6 h and 210 ± 10 °C for 3 and 6 h. After each D-F process, a 400 mL sample was collected in standard amber glass vials to monitor oil degradation and track changes in oxidized oil under various conditions (oil variety, frying time, frying temperature, and added natural antioxidants). The collected oil samples were stored at 5 °C in dark conditions to prevent further oxidation before analysis. The sensory profile, pigments, antioxidant potential, and NIR spectroscopy of the oils were evaluated for both control (non-fried) and fried samples across all oil categories.

### 2.4. Evaluation of Sensory Attributes by Panel

This study assessed 19 sensory descriptors as reference analyses in various olive oils compared to sunflower oils under different D-F conditions. Among these, 15 negative attributes were evaluated, including fusty/muddy sediment, musty/humid/earthy, winey/vinegary/acid/sour, frostbitten olives (wet wood), rancid, metallic, dry hay, grubby, rough, brine, heated or burnt, vegetable water, esparto, cucumber, and greasy. Additionally, four positive attributes—green fruity, ripe fruity, bitter, and pungency—were analyzed. However, only five descriptors (one defect and four positive markers)—rancid, green fruity, ripe fruity, bitter, and pungent—were successfully identified as key indicators of sensory quality across the different olive oil categories under D-F conditions with multivariate analyses. The remaining negative attributes, including musty/humid/earthy, metallic, dry hay, grubby, rough, brine, heated or burnt, vegetable water, esparto, cucumber, and greasy, were not detected by the panelists in any olive oil categories or sunflower oils, regardless of frying status. The oil samples were classified based on the intensity of their defects and positive attributes, with evaluations carried out by a panel of trained assessors. The panel, consisting of 10 trained members and led by a panel leader, used the official procedures described in Commission Regulation (EEC) No 2568/91 [32], with later modifications [33,34], and the procedure of International Olive Council [7] to perform organoleptic assessments of the olive oil samples. The panelists were equipped with narrow-mouthed glasses to score the odor, smell, and taste of the samples before filling out evaluation forms for positive and negative attributes. Negative attributes included fusty, musty, winey, rancid, and others, while positive attributes included green fruity, ripe fruity, bitter, and pungency, all assessed on a 10 cm unstructured line scale. Each sample was analyzed in triplicate, and the mean value was subsequently used as the response variable for calibration modeling against the NIR dataset.

### 2.5. UV–Visible Spectrophotometric Analysis of Carotenoids, Chlorophyll, and Antioxidant Activity

The total carotenoid and chlorophyll contents in the EVOO, other olive oil categories, and sunflower oils were determined using absorption spectrophotometry with a UV–Vis spectrophotometer (model 8453 Hewlett Packard, Waldbronn, Germany), following the method of Cayuela et al. [23]. Measurements were taken at 472 nm and 670 nm for carotenoids and chlorophylls, respectively, using pigment extracts in cyclohexane. In brief, 7.5 g of the oil sample was weighed and dissolved in 25 mL of cyclohexane in a volumetric flask, then stirred to ensure homogenization. The sample was subsequently analyzed using a spectrophotometer. The results are expressed in mg/kg of oil, with all samples measured in triplicate.

The antioxidant capacity of the EVOO, low-quality olive oil, and sunflower oils was evaluated using the DPPH assay [35]. A 0.15 mM DPPH methanolic solution was prepared, and 1 mL of EVOO methanolic extract was mixed with 0.5 mL of DPPH, stirred, and incubated for 30 min at room temperature. Absorbance was measured at 517 nm against a blank. Ascorbic acid was used as the standard antioxidant compound. All measurements were conducted in triplicate.

(1) $$DPPH\;scavenging\;activity\left(\%\right)=\frac{A0-A1}{A0}\times 100\;$$

where A0 = absorbance of the control and A1 = absorbance of the sample

### 2.6. NIR Spectroscopy Acquisition

A total of 426 spectra were collected from 142 oil samples, each measured in triplicate using near-infrared (NIR) spectroscopy. Before spectral acquisition, the samples were centrifuged at 20,000 rpm for 30 min (Sorvall RC-6 Plus, Osterode, Germany) to eliminate particles and dispersed water droplets, thereby minimizing light scattering effects. NIR spectra were then obtained with a Foss NIRSystems 5000 spectrophotometer (Foss NIRSystems, Silver Spring, MD, USA) equipped with a thermostated liquid analyzer module and a Suprasil quartz flow cell. The acquisition settings included a 10 mm optical path length, a wavelength range of 1100–2498 nm, a spectral resolution of 2 nm, and 32 scans per spectrum.

### 2.7. Data Analyses and Chemometrics

The data obtained from the conventional analyses were calculated as mean ± standard deviation (SD) based on three replicates and calculated using SPSS software (version 28, IBM SPSS Statistics, Chicago, IL, USA). These mean values were then used for further multivariate data analyses. The composition of pigments (carotenoids and chlorophyll), DPPH radical scavenging activity, and sensory markers (rancid, fruity green, fruity ripe, bitter, and pungent) in the non-fried, fried, supplemented, and non-supplemented olive oils—as well as in sunflower oil and high-oleic sunflower oil—was predicted using multivariate techniques.

The NIR dataset was processed with V-PARVUS chemometric software by applying the SELECT algorithm for variable selection, followed by OLS regression (SELECT–OLS) based on the selected spectral variables to develop robust multivariate calibration models. To improve data quality, standard normal variate (SNV) preprocessing was applied. Model development and variable selection were performed using the V-PARVUS software (version PARVUS2011, Michele Forina, Genoa, Italy). In the case of regression problems, SELECT–OLS first selects the predictor with the largest correlation with the response. The SELECT algorithm ranks wavelengths by correlation weights, determining the selection order and contribution of each variable to the model. Using an iterative approach, it first selects the most correlated variable, then reduces redundancy by decorrelating remaining predictors, enhancing interpretability and accuracy. By integrating variable selection into validation, SELECT minimizes overfitting and ensures reliable predictions. Additionally, SELECT ranks variables by selection frequency and order across multiple cross-validation cycles, further refining regression models for an improved predictive performance [24,36]. Therefore, the SELECT algorithm was first applied to identify a subset of decorrelated, significant variables, optimizing regression models for pigments, antioxidant activity, and sensory attributes. In NIR spectroscopy, SELECT iteratively selected the most influential predictor and removed redundancy until a predefined threshold was met, enhancing predictive accuracy by eliminating irrelevant variables. After variable selection with SELECT, full-spectrum OLS models were developed to quantify carotenoids, chlorophyll, DPPH radical scavenging activity, and sensory markers.

In addition, OLS regression models were evaluated using leave-one-out (LOO) cross-validation metrics, including residual standard deviation and mean prediction error. The most robust model was identified based on the lowest LOO mean prediction error. Additional performance metrics included standard deviation of error (measuring prediction variability), mean absolute error (MAE), and multiple correlation coefficient (R), which account for explained variance while considering the number of predictors. To prevent overfitting, the optimal number of predictors in the SELECT–OLS model was determined through rigorous validation, ensuring an unbiased predictive accuracy.

## 3. Results and Discussion

### 3.1. Spectral Analysis of Oil Samples

**Figure 1:**
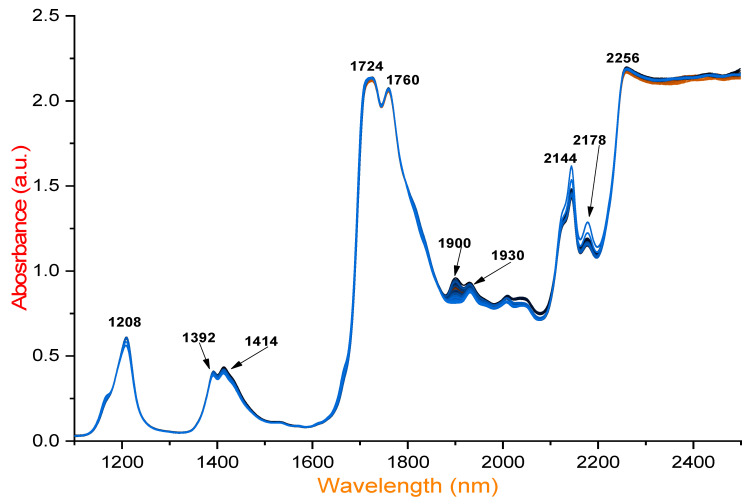
NIR spectra of different varieties of EVOO, mixed EVOO/VOO with ROO olive oils, pomace olive oil, sunflower oils, and sunflower oil with high oleic acid under frying conditions, compared to non-fried and non-supplemented olive oil with HTyr.

NIR spectroscopy was employed to assess the quality of EVOO, other low-quality olive oils, and different sunflower oils before and after deep-frying and with or without exogenous supplementation with HTyr extract, identifying the key absorption bands associated with various chemical components. A total of 426 spectra were recorded from 142 different samples. The findings reveal that prominent peaks at 1208 nm are particularly useful for evaluating quality parameters, including free fatty acid content. Additionally, significant absorption at 1392 nm corresponds to O–H combination bands, which are valuable for quality assessment (Figure 1). The spectral range of 1350–1570 nm proves effective in differentiating olive oils, aiding in authentication and quality control by distinguishing original olive oil and detecting primary oxidation compounds. Higher absorbance at 1414 nm, attributed to overtones of C–H and O–H bonds, is also identified in this study. Moreover, the NIR spectra exhibit significant absorption at 1724 nm due to the first overtone of C–H vibrations. Similarly, absorption at 1760 nm is linked to the first overtone of C–H vibrations and lipid oxidation, enabling the detection of primary oxidation products. Obviously, high absorbance at 1900 nm corresponds to oxidation and degradation processes.

In sunflower oil, the strong absorbance near 2144 nm is linked to C–H stretching, signaling the formation of aldehydes and ketones due to lipid degradation. This wavelength primarily corresponds to the C–H stretching vibrations of cis-unsaturated fatty acids, making it a key indicator of cis double bonds in fatty acid chains. Notably, the intense absorption at 2145 nm can overshadow peaks associated with saturated and trans fatty acids, typically found around 2128 and 2131 nm, respectively. Therefore, absorption at 2145 nm serves as a distinct marker for detecting and analyzing cis-unsaturation in fatty acids using NIR spectroscopy.

A similar absorption peak at 2178 nm is observed in sunflower oil, high-oleic sunflower oil, and pomace olive oil, also linked to C=O stretching and lipid degradation. These oils exhibit a lower stability compared to EVOO, EVOO blended with refined olive oil (ROO), and virgin olive oil (VOO) blended with ROO. Additionally, significant absorption at 2256 nm varies among the samples and is found to be useful for distinguishing oxidation stability. This peak, associated with O–H and C–H combination bands, is relevant for assessing hydrolysis and secondary oxidation. Furthermore, Table 1 presents wavelength ranges (nm), associated compounds, and their significance in olive oil analysis and oxidation detection. Overall, the spectral range of 1700–2500 nm strongly correlates with lipid oxidation and hydrolytic degradation [37,38,39,40]. NIR spectroscopy has proven to be a powerful tool for classifying EVOO based on spectral variations. These findings highlight its broad applicability in ensuring EVOO authenticity, thermal stability, freshness, and overall quality.

### 3.2. Performance of SELECT–OLS for Carotenoid Quantification

To quantify the carotenoid levels of several olive oil and sunflower types using NIR spectra, prediction models are constructed by integrating SELECT–OLS with various variable selection. The most effective subset of wavelengths is illustrated in Table 2 part A. From the 700 variables recorded in the NIR system, SELECT–OLS identifies only 30 latent key variables for predicting carotenoid content. The findings illustrate that 1904 nm is the first-selected wavelength, with the highest correlation coefficient (0.72), followed by 1888 nm (0.21). The 1800–2000 nm region in NIR spectroscopy is crucial for assessing olive oil oxidation and quality. Absorption near 1900 nm indicates O–H stretching in hydroperoxides (primary oxidation products). The first overtone of C–H stretching from unsaturated fatty acids also appears here, with decreasing absorption indicating oxidative degradation. Higher absorption in the 1900–1950 nm range suggests increased oxidation, making this region vital for monitoring rancidity and oxidative stability in oil samples [16].

The current findings indicate that before deep-frying, the olive oil samples exhibit higher absorption at 1900 nm, suggesting a high concentration of primary oxidation compounds. In contrast, sunflower oil and olive oil subjected to high temperatures for extended periods display lower absorbance, indicating the presence of higher levels of secondary oxidation compounds due to the degradation of primary oxidation products. Similarly, previous studies by Naz et al. [47] and Zhang et al. [48] have demonstrated that as frying progresses, primary oxidation compounds gradually convert into secondary oxidation products.

**Figure 2:**
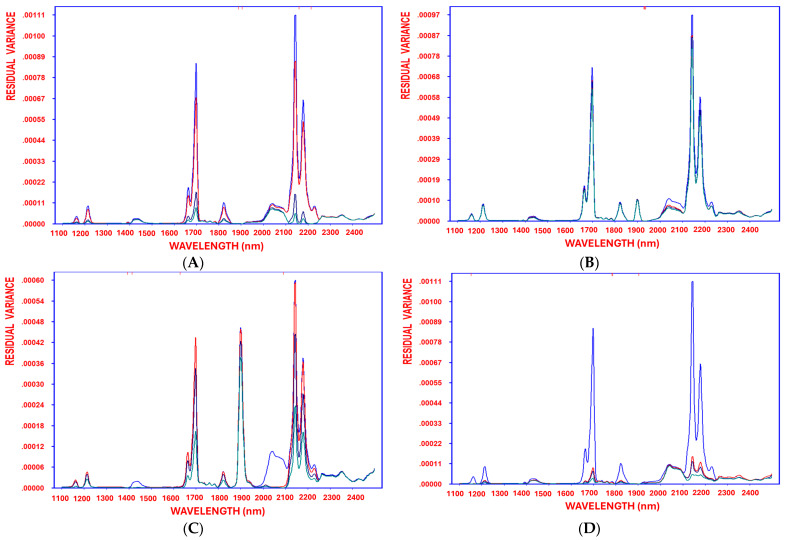
Residual variance of the variables decorrelated by SELECT after 1, 2, 3, and 4 selections (blue, red, turquoise, and green, respectively), obtained when working with NIR spectra after applying standard normal variate (SNV) as preprocessing technique, using carotenoids (**A**), chlorophyll (**B**), antioxidant activity (**C**), and rancidity (**D**) as response variables.

Table 2 part B shows the performance metrics for the quantification of carotenoids, allowing for an easy visual evaluation of the models’ performance. The model exhibits a strong fit, with R = 0.96 and standard deviation of the error = 1.54, confirming its robustness. Leave-one-out (LOO) cross-validation metrics further support its predictive performance, demonstrating a low mean prediction error (1.36). The SELECT algorithm identifies a distinct set of 30 key wavelengths to predict the carotenoids. The current results suggests that critical spectral oxidation features may vary based on factors such as EVOO variety, deep-frying conditions (temperature and time), and carotenoid stability during deep frying. The differences in the selected wavelengths correspond to O–H and C–H combination bands, which are associated with secondary lipid degradation. Additionally, the steady reduction in residual variance, from the initial stage before variable selection through each decorrelation cycle until the model reaches optimal complexity, demonstrates the method’s effectiveness in minimizing residual variance (Figure 2A). The current OLS model demonstrates a greater robustness compared to a recent model in another study using partial least squares regression (PLSR) to predict carotenoids. This PLSR model achieved a coefficient of determination (Rc^2^ = 0.62), as reported by Cayuela et al. [44], indicating a moderate correlation between predicted and reference values. While the predictive performance achieved by SELECT–OLS in the present research indicates a high reliability and accuracy, further validation may be required to confirm its effectiveness.

### 3.3. Performance of SELECT–OLS for Chlorophyll Quantification

Chlorophylls are essential pigments in olive oil, significantly influencing its color and oxidative stability. They impart a green hue to the oil, with concentrations varying based on olive variety, olive cultivar, ripeness stage, and processing methods. While chlorophylls can act as pro-oxidants under light exposure, leading to photo-oxidation, they may also exhibit antioxidant properties in the absence of light, contributing to the oil’s stability [49]. Therefore, chlorophylls play a dual role in determining olive oil’s color and oxidative stability.

Out of the 700 spectral variables recorded by the NIR system, the SELECT–OLS method identified 30 key wavelengths for predicting chlorophyll content across various oil types (Table S1A). The most significant predictor was 1932 nm, selected first with the highest weight (0.53), followed by 1936 nm (0.37). Table S1B summarizes the model’s performance metrics, offering a clear view of its predictive strength. The model achieved a strong correlation (R = 0.95) and a standard error of 4.31, confirming its robustness. Leave-one-out (LOO) cross-validation further supported its reliability, with a low mean prediction error of 3.88.

The SELECT algorithm pinpointed a unique set of 30 influential wavelengths, reflecting variations in spectral oxidation features driven by factors such as EVOO variety, refined olive oil type, sunflower oil frying conditions (temperature and time), and carotenoid degradation during frying. The initial two selected wavelengths were associated with the C=O stretching vibrations of aldehydes and ketones—compounds indicative of secondary lipid oxidation. The results also show that oils fried at lower temperatures for shorter durations exhibited reduced levels of secondary oxidation products.

Additionally, Figure 2B illustrates the progressive reduction in residual variance throughout the variable selection process. This decrease—from the initial model stage through each decorrelation cycle to the point of optimal model complexity—demonstrates the method’s efficiency in minimizing residual variance. Compared to recent PLSR-based models for chlorophyll prediction, the current model exhibits a superior robustness. For instance, the model by Cayuela et al. [44] reported an Rc^2^ of 0.56, indicating moderate predictive power, whereas the performance of the SELECT–OLS model in this study shows a substantially higher accuracy and reliability.

### 3.4. Performance of SELECT–OLS for Antioxidant Activity Quantification

The results show that SELECT–OLS identified 30 key variables from the 700 recorded in NIR spectroscopy (Table S2A) to quantify antioxidant activity across different oil categories, with 1394 nm as the first-selected wavelength (correlation coefficient = 0.71), followed by 1414 nm (correlation coefficient = 0.24). The model demonstrated a strong fit (R = 0.96 and standard error = 7.17), confirming its robustness, while leave-one-out (LOO) cross-validation validated its predictive accuracy with a low mean prediction error of 6.44 (Table S2B). The SELECT algorithm identified 26 critical wavelength markers, highlighting spectral oxidation variations influenced by EVOO variety, deep-frying conditions (temperature and time), and the stability of antioxidant compounds, e.g., polyphenols, during frying. Differences in the first two selected wavelengths corresponded to overtones of C–H and O–H bonds, linked to oil differentiation [41,42], where high absorbance at 1394 nm was detected in the supplemented oil samples before deep-frying, particularly in the HTyr-supplemented olive oils, indicating that NIR spectroscopy can easily distinguish supplemented oils due to trace compounds originating from the extract. Additionally, the continuous reduction in residual variance—from the initial stage before variable selection through each decorrelation cycle until the model reached optimal complexity—demonstrates the method’s efficiency in minimizing residual variance (Figure 2C).

### 3.5. Performance of SELECT–OLS for Rancidity Quantification

The current findings reveal that SELECT–OLS identified 29 markers from 700 variables for the quantification of rancidity sensory defects (Table S3A). The first-order selection ranked 1902 nm with the highest weight (0.70), followed by 1152 nm (0.21). The model demonstrated a strong fit (R = 0.95), confirming its robustness. LOO cross-validation metrics indicated a strong predictive performance, with a low mean prediction error of 0.88 (Table S3B). Furthermore, the SELECT algorithm identified a distinct set of wavelengths, highlighting that spectral oxidation features may vary based on EVOO variety, deep-frying conditions (temperature and/or time), and rancid attribute observation by panelists. Furthermore, the steady reduction in residual variance—from the initial stage before variable selection through each decorrelation cycle until the model reached optimal complexity—demonstrates the method’s effectiveness in minimizing residual variance (Figure 2D).

Low absorbance at 1902 nm (the first selected variable) was observed, particularly for EVOOs of the Picual, Arbequina, Empeltre, and Cornicabra varieties, as well as for refined olive oil, sunflower oil, and high-oleic sunflower oil, especially at low temperatures and short frying times, suggesting lower levels of rancidity. The low rancidity values for sunflower oils, as perceived by the panelists, and the reduced absorbance at this wavelength can be attributed to their higher smoke point compared to olive oil. Sunflower oil generally has a higher smoke point than EVOO, making it more suitable for high-temperature cooking methods like deep-frying, with smoke points from approximately 227 °C to 232 °C for sunflower oil and from 177 °C to 210 °C for EVOO. These values indicate that sunflower oil can withstand higher cooking temperatures before reaching its smoke point. However, its stability during frying is influenced not only by its smoke point, but also by its oxidative stability, degree of unsaturation, contents of free fatty acids and antioxidants, and external factors such as the length of heating, temperature and surface-to-oil volume ratio, and fatty acid composition [4,50]. The results also show that high-oleic sunflower oil exhibited a greater stability than regular sunflower oil due to its higher oleic acid content. Meanwhile, EVOO, despite its relatively lower smoke point, contains natural antioxidants, particularly in supplemented varieties, and has a favorable monounsaturated fat profile, contributing to its stability under deep-frying conditions. Therefore, while sunflower oil’s higher smoke point makes it ideal for high-temperature frying, EVOO remains a stable and healthful option due to its unique sensorial attributes and natural antioxidants [8].

As deep-frying time and temperature increased, rancidity values rose significantly. Additionally, absorption at 1152 nm (the second selected variable), primarily associated with C–H second overtone stretching vibrations in lipids and fatty acids, exhibited distinct variations among the studied oils. Sunflower oils and olive oils fried under prolonged conditions showed higher absorbance, indicating a greater susceptibility to thermal degradation, while EVOOs, particularly the Manzanilla, Koroneiki, and Arbosana varieties, displayed lower absorbance and greater thermal stability. The differences in absorbance at this wavelength among vegetable oils suggest structural modifications in fatty acids due to oxidation, frying conditions, or the presence of natural antioxidants. Therefore, EVOO varieties and cultivars differ in their oxidative stability due to variations in polyphenol content, natural antioxidants, and oleic acid composition, which are critical factors to consider before using them for deep frying [4,50].

The dynamic changes observed in the oils were likely due to various factors affecting thermal stability, the formation of off-flavors, and the generation of rancid molecules. However, as perceived by the panelists, EVOO varieties, particularly when fried at a lower temperature (170 °C) for a shorter time (3 h) with continuous deep-frying, exhibited lower rancidity values, along with the perception of some desirable sensory attributes. On the other hand, sunflower oil and refined olive oil also received low rancidity scores under the same conditions, but they lacked key sensorial attributes such as fruitiness, bitterness, and pungency, which are characteristic of EVOO. This suggests that EVOO is a better choice for frying, offering both health benefits and bioactive compounds while maintaining superior sensory properties.

### 3.6. Performance of SELECT–OLS for Fruity Green Quantification

The current findings show that the SELECT–OLS algorithm identified 30 key markers from the 700 NIR spectral variables for the quantification of fruity green sensory attributes (Table S4A). The wavelength at 1892 nm was ranked first with the highest weight (0.53), followed by 1908 nm (0.19). The model demonstrated a strong fit with a correlation coefficient of R = 0.88, confirming its robustness. Leave-one-out (LOO) cross-validation further supported the model’s predictive accuracy, yielding a low mean prediction error of 0.82 (Table S4B).

The SELECT algorithm identified a distinct set of wavelength predictors, suggesting that variations in spectral oxidation features were influenced by factors such as EVOO variety, frying conditions (temperature and/or time), and the perception of green sensory attributes by the panelists.

Low absorbance at 1892 nm (the first selected variable) was observed, particularly in fried olive oils subjected to minimal continuous deep-frying stress (i.e., lower time and temperature), suggesting a high thermal stability and the retention of fruity attributes. In contrast, pomace olive oil, refined olive oil, and sunflower oils exhibited a lack of green sensory attributes due to the refining process. Similarly, most EVOO varieties exposed to prolonged deep-frying stress showed a decrease in or complete loss of these desirable properties.

**Figure 3:**
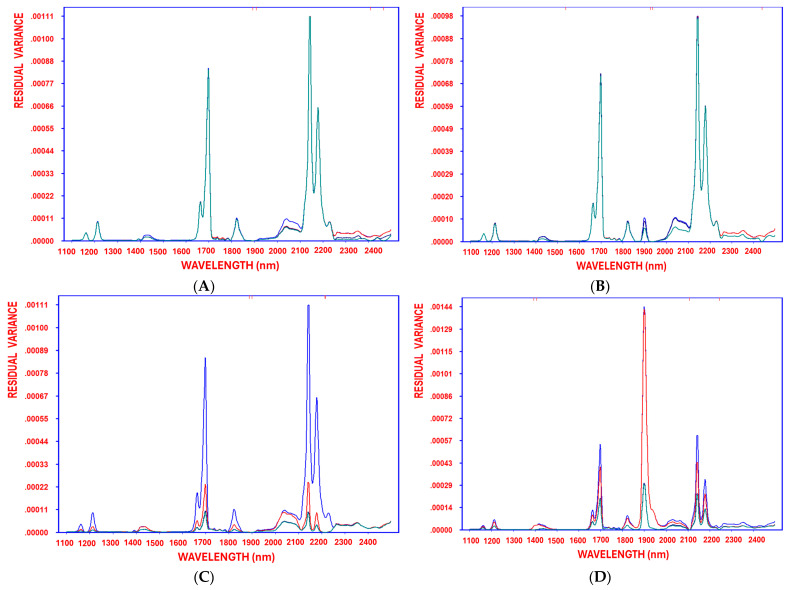
Residual variance of the variables decorrelated by SELECT after 1, 2, 3, and 4 selections (blue, red, turquoise, and green, respectively), obtained when working with NIR spectra after applying standard normal variate (SNV) as preprocessing approach, using fruity green (**A**), fruity ripe (**B**), bitter (**C**), and pungent (**D**) as response variables.

As deep-frying time/temperature increased, fruity values significantly decreased. Furthermore, the steady reduction in residual variance—from the initial stage before variable selection through each decorrelation cycle until the model reached optimal complexity—demonstrates the method’s effectiveness in minimizing residual variance (Figure 3A). The SELECT–OLS model shows a slightly higher robustness (R^2^ = 0.88) than recent PLSR models (R^2^ = 0.87) in predicting the fruity attributes, as reported by Inarejos-García [9]. These findings reinforce NIR spectroscopy combined with chemometrics as an effective tool for the real-time assessment of olive oil sensory attributes.

### 3.7. Performance of SELECT–OLS for Fruity Ripe Quantification

SELECT–OLS effectively extracted 30 informative wavelengths from the 700 recorded spectral variables for modeling fruity ripe sensory attributes (Table S5A). The most influential wavelengths were 1936 nm (weight = 0.40) and 1926 nm (weight = 0.25). The resulting model showed a solid fit (R = 0.87) and demonstrated a reliable predictive ability, as confirmed by leave-one-out cross-validation with a low mean prediction error of 0.72 (Table S5B). The selected wavelengths reflect differences in spectral oxidation patterns, likely influenced by factors such as EVOO cultivar, frying temperature and duration, and panelist perceptions of ripe attributes.

High absorbance at 1936 nm (the first selected variable) may be linked to primary oxidation compounds, such as hydroperoxides, which form in the early stages of oil degradation. In this regard, high absorbance was particularly observed for both non-fried supplemented and non-fried non-supplemented EVOOs, suggesting pronounced ripeness attributes. In contrast, sunflower oil exhibited low absorbance values, indicative of advanced secondary degradation and oxidation, and, thus, a lack of the desirable fruity ripe characteristics. Moreover, as deep-frying time/temperature increased, ripeness values significantly decreased. Furthermore, the steady reduction in residual variance—from the initial stage before variable selection through each decorrelation cycle until the model reached optimal complexity—demonstrates the method’s effectiveness in minimizing residual variance (Figure 3B).

### 3.8. Performance of SELECT–OLS for Bitterness Quantification

SELECT–OLS identified 30 significant wavelengths out of 700 for predicting fruity green sensory attributes (Table S6A). The highest weight was assigned to 1900 nm (0.55), followed by 2216 nm (0.16). The model demonstrated a strong fit (R = 0.88), and leave-one-out cross-validation confirmed its predictive reliability, yielding a low mean prediction error of 0.73 (Table S6B). The selected wavelengths highlight variability in spectral oxidation features, which appear to be influenced by EVOO variety, frying conditions (temperature and time), and the panelists’ perception of bitterness.

Notably, the highest absorbance at 1900 nm—the most significant variable—was observed in both non-fried supplemented and non-fried non-supplemented EVOOs. Absorption at this wavelength, typically associated with the first overtone of O–H stretching vibrations, primarily reflects the water content in a sample. Increased absorbance here may imply a higher presence of water or early-stage oxidation products (such as hydroperoxides), potentially signaling the onset of degradation or hydrolytic reactions. This effect may be due to fortification processes involving tyrosol or centrifugation procedures that generate primary oxidation compounds; however, the natural antioxidants present in EVOO help to delay the progression of rancidity. Conversely, both supplemented and non-supplemented olive oils before deep-frying exhibited strong bitterness attributes. Overall, while the interactive dynamics in olive oil are complex, the EVOO samples ultimately demonstrated considerable thermal stability combined with highly favorable sensory characteristics. In contrast, sunflower oil and refined olive oil, as well as EVOO subjected to prolonged deep-frying, did not exhibit bitterness, as perceived by the panelists. Furthermore, as deep-frying time and temperature increased, bitterness levels declined significantly [8]. Moreover, the progressive reduction in residual variance, from the initial stage before variable selection through each decorrelation cycle to the model reaching optimal complexity, demonstrates the method’s efficiency in minimizing residual variance (Figure 3C). The SELECT–OLS model in the current study shows a higher robustness (R^2^ = 0.88) than recent PLSR models (R^2^ = 0.85) in predicting the bitterness attributes, as reported by Inarejos-García [9].

Additionally, the low R-values observed for some sensory attributes when compared to carotenoid (Table 2), chlorophyll (Table S1), and antioxidant activity (Table S2) measurements may be attributed to the instantaneous and more precise nature of these chemical analyses, which generally have a lower error than human sensory evaluations. Therefore, we recommend further studies involving EVOOs from various origins and conducted by panels with greater experience.

### 3.9. Performance of SELECT–OLS for Pungency Quantification

The results indicate that SELECT–OLS identified 30 key markers from 700 variables for the quantification of pungent sensorial markers (Table S7A), with the highest correlation coefficients assigned to 2108 nm (0.47) and 2244 nm (0.30). The model exhibited an effective fit (R = 0.86 and standard deviation of the error = 0.91), while LOO cross-validation metrics confirmed its robust predictive performance, with a low mean prediction error of 0.86 (Table S7B). The SELECT algorithm identified a distinct set of wavelengths, suggesting that spectral oxidation features vary based on EVOO variety, deep-frying conditions, and panelists’ perception of pungency.

Absorption at 2108 nm is primarily associated with combination bands involving C–H stretching and bending vibrations in fatty acid chains. In edible oils, this wavelength provides insights into molecular alterations resulting from oxidation or thermal degradation [51]. Specifically, changes in this band can indicate variations in the degree of unsaturation and the formation of oxidation products, thereby affecting oil quality and stability. Monitoring this band can, thus, complement other key wavelengths in assessing the overall condition of oil during deep frying. Furthermore, the highest absorbance at 2108 nm—the most significant variable—was observed in fried low-quality oils such as pomace olive oil, high-oleic sunflower oil, and conventional sunflower oil, indicating high rancidity and reduced pungency. In contrast, EVOOs, including varieties such as Manzanilla, Arbosana, Picual, Royuella, and Koroneiki, exhibited lower absorbance values at this band, reflecting minimal oxidation and a more pronounced perception of pungency. This observation was corroborated by sensory panel evaluations, which consistently rated EVOOs higher than refined olive oil and sunflower oils in terms of desirable sensory properties. As this band corresponds to secondary oxidation compounds like aldehydes, these findings suggest that EVOOs are more stable under thermal processing than refined olive oils and sunflower oils due to their higher polyphenol content and natural antioxidants. Notably, a high pungency is indicative of high-quality oils with elevated polyphenol levels that enhance stability, while deep-frying under extended time and temperature conditions leads to a significant decline in bitterness levels. Moreover, the progressive reduction in residual variance, from the initial stage before variable selection through each decorrelation cycle to the model reaching optimal complexity, demonstrates the method’s efficiency in minimizing residual variance (Figure 3D).

In sum, these findings confirm the robustness and versatility of SELECT–OLS as an effective feature selection and correction method for predicting pigment content, antioxidant capacity, and sensory attributes across various olive oil categories—both fried and non-fried and supplemented or non-supplemented with exogenous polyphenols—as well as different sunflower oil types. By systematically reducing dimensionality while maintaining a high predictive performance, SELECT–OLS optimizes model complexity, minimizes residual variance, and enhances the accuracy of chemical and sensory calibration models in complex oil matrices. Its consistent performance across various chemical and sensory markers further underscores its reliability for spectral data analysis and qualitative modeling. The purpose of this study was achieved by developing regression models that predict the pigment, antioxidant, and sensory properties of oil samples based on measured spectral data. In SELECT–OLS, prediction is performed by transforming inter-correlated variables into a set of independent factors called latent variables (LVs), which capture the maximum covariance between the spectral information and the response variables. These statistically independent LVs encompass all the relevant information needed to achieve stable predictions.

This study also highlights the potential of NIRS as a predictive and quantitative tool for assessing olive oil quality, particularly regarding pigment content, DPPH scavenging activity, and sensory parameters. Moreover, the findings emphasize the importance of portability in olive oil and sunflower oil quality control. The combination of NIRS with SELECT–OLS regression modeling offers a rapid, sustainable, low-cost, non-destructive assessment method that can be conveniently deployed in the food industry or at restaurants, reinforcing the reliability of these methodologies for future applications. Overall, this research advances quality assessment for both olive and sunflower oils by demonstrating how variable selection methods enhance NIRS predictive performance for pigments, antioxidants, and sensory characteristics, while also underscoring the potential of NIRS devices for fast and accurate quality evaluation.

Several studies support and complement the findings presented here. For instance, Páscoa et al. [52] demonstrated the efficacy of NIR spectroscopy combined with multivariate regression to predict the composition of olive pomace samples. Their partial least squares (PLS) models yielded high coefficients of determination for prediction (R^2^_*p* > 0.9) and range error ratios (RER > 12). However, PLS models for antioxidant activity showed less satisfactory results. For PLS-discriminant analysis (PLS-DA) models, the validation accuracies were 82%, 90%, and 61% for DPPH scavenging ability, total phenolic content (TPC), and ferric-reducing antioxidant power (FRAP), respectively.

Mehany et al. [22] employed feature selection techniques to enhance the accuracy of phenolic content prediction in various olive oils, consistent with our application of the SELECT–OLS method for dimensionality reduction and model optimization. The SELECT algorithm effectively reduced redundant wavelengths, selecting up to 30 key predictors from an initial 700, improving model simplicity and accuracy. Their models achieved high correlation coefficients (R = 0.91–0.98) for multiple phenolic compounds, including hydroxytyrosol (HTyr), tyrosol (Tyr), caffeic acid, oleocanthal, oleacein, and total phenolic content (TPC). The predictive accuracy was strong, with low leave-one-out mean prediction errors (LOOMPEs), such as 15.69 mg/kg for HTyr and 82.38 mg/kg for TPC. The variable number of selected predictors across compounds demonstrates the flexibility of SELECT–OLS to accommodate differing spectral signatures. These models performed robustly across diverse oil types, including supplemented and non-supplemented samples and samples under various deep-frying conditions (3–6 h at 170–210 °C). Overall, the SELECT–OLS approach offers a rapid, non-destructive, eco-friendly, and cost-effective solution for routine phenolic content evaluation in edible oils, facilitating online and routine quality control.

Poorly emphasized but critical aspects for a sustainable olive oil system include replacing traditional chemical analysis methods and designing quality parameters for the final product. NIRS plays a pivotal role here. Recent studies comparing different NIRS systems for predicting the moisture, oil content, soluble solids, total phenolic content, and antioxidant activity of intact olive drupes have shown promising results. Fourier transform (FT)–NIR spectrometers equipped with integrating spheres and fiber-optic probes, alongside Vis/NIR handheld devices, produced encouraging predictive models (0.64 < R^2^_pred < 0.84) with minimal biases. While FT–NIR models are recommended for online or inline applications in oil mills to improve quality design, portable Vis/NIR devices provide a cost-effective and practical option for preliminary quality evaluation directly in the field [53].

The oxidative stability index (OSI) is essential for assessing the commercial, nutritional, and sensory qualities of EVOOs. NIRS offers a rapid, cost-effective alternative to traditional methods like Rancimat for OSI evaluation. A recent study developed a robust global NIRS model to predict the OSI in EVOO and compared it with cultivar-specific models for key Spanish varieties, including ‘Picual’, ‘Arbequina’, and ‘Sikitita’—a new cultivar for commercial hedgerow systems. Using spectral data from 1100 to 2500 nm and analyzing 939 global samples, they developed partial least squares regression models that demonstrated a strong performance, with the global model outperforming individual yearly models due to its incorporation of broader variability. Log-transformed OSI data further enhanced prediction accuracy. Additionally, linear discriminant analysis on NIRS spectra from five cultivars (‘Arbequina’, ‘Picual’, ‘Koroneiki’, ‘Sikitita’, and ‘Arbosana’) with 254 samples achieved a 96% accuracy in differentiating monovarietal EVOO samples. These findings underscore the versatility of NIRS for OSI modeling and cultivar discrimination, proving valuable for breeding programs and quality control [54].

Beyond olive oils, NIR spectroscopy has been employed to predict shelf life in other agricultural products, such as sweet corn. Using an advanced accelerated shelf-life testing model combined with multivariate analysis, researchers integrated key postharvest quality parameters—including weight loss, dry matter, hardness, total soluble solids, respiration rate, and sugar contents—into a comprehensive corn quality index. Spectral data collected from samples stored at 4, 13, and 25 °C over 17 days were analyzed using kinetic models (Arrhenius and PLS regression). The NIR-based predictions closely matched actual shelf-life measurements, demonstrating that spectral data can non-destructively and rapidly estimate sweet corn quality and shelf life. This approach offers a flexible and effective tool for real-time postharvest quality management [55].

## 4. Conclusions

The findings of this study demonstrate that NIR spectroscopy, combined with multivariate calibration and the SELECT–OLS method, provides a robust and effective approach for quantifying key quality parameters in olive and sunflower oils. Our reduced-spectrum regression models—utilizing only a limited number of carefully selected wavelengths—successfully quantified pigments (such as carotenoids and chlorophyll), antioxidant capacity, and key sensory attributes (including rancidity, fruity green, fruity ripe, bitter, and pungent) across various vegetable oil types. These models maintained a high predictive performance even under challenging conditions, such as deep-frying (3–6 h at 170–210 °C), while keeping model complexity to a minimum. Importantly, the high correlation coefficients and low prediction errors achieved confirm the feasibility of this foodomic approach for rapid, green, and non-destructive quality assessment. Commercially, these insights are significant: implementing NIR devices integrated with SELECT–OLS regression could revolutionize quality control in the edible oil industry. Such systems would enable real-time, on-site monitoring during production and processing, drastically reducing the reliance on time-consuming traditional chemical analyses and subjective sensory evaluations. This not only enhances operational efficiency and cost-effectiveness, but also supports the consistent production of high-quality oils that meet stringent sensory and nutritional standards. Additionally, the model developed in the current study is robust in quantifying not only chemical and sensory markers, but also thermal stability under various continuous deep-frying conditions. Ultimately, the integration of these advanced spectral analysis techniques into commercial quality assurance protocols promises to elevate product quality and ensure greater consumer satisfaction in the marketplace. It is recommended that future studies utilize extra virgin olive oils from various locations and employ taste test panels with more experienced tasters to achieve even better and more consistent results.

## Figures and Tables

**Table 1:** Wavelength ranges (nm), functional groups, associated compounds, and their significance in olive oil analysis and oxidation detection.

Wavelength (nm)	Functional Groups	Assignment	Significance in Oil Analysis and Oxidation Detection	Source
1100–1150	–CH_3_	C–H stretching in lipids	Triglycerides and oil purity detection	[37,38,39,40]
1167	–CH_3_	C–H stretching 2nd overtone	Triglycerides and oil purity detection	[41,42]
1208	–CH_2_	C–H stretching 2nd overtone	Free fatty acid estimation	[41,43]
1220	HC=CH–	C–H stretching 2nd overtone	Unsaturation levels assessment, which are critical for nutritional value and oxidative stability	[44]
1392	–CH_3_	2C–H stretching + C–H deformation	Oils differentiation and fatty acid characterization	[41,42]
1414	–OH	O–H stretching	Oils differentiation and monitoring oil degradation	[41,45]
1724	–CH_2_, –CH_3_, =CH_2_	C–H 1st overtone	Primary oxidation products detection, degradation and oxidation assessment	[41,42]
1760	–CH_2_, –CH_3_, =CH_2_	C–H 1st overtone	Primary oxidation products detection, degradation and oxidation assessment	[46]
1900	O–H (Hydroxyl) group	O–H stretching	Deformation of hydroperoxides (ROOH) and assessment of oxidative stability and quality control	[37,38,39,40]
1930–1950	O–H (Hydroxyl) group	C=O stretching	Secondary oxidation monitoring and assessment of aldehydes and ketones and useful for quality assessment of the oil	[37,38,39,40]
2022	–COOR	C–H stretching + C=O stretching	Detection of oxidation and rancidity and olive oil quality assessment	[41,42]
2049	–COOR	C–H stretching + C=O stretching	Detection of oxidative changes and degradation	[41,42]
2144	HC=CH–	C–H stretching + C=C stretching	Aldehydes and ketones formation from lipid degradation	[41,42]
2200–2300	C=O, −CH_2_–, and –CH_3_–	O–H and C–H combination bands	Hydrolysis and secondary oxidation assessment	[37,38,39,40]
2350–2500	–CH_2_– and –CH=CH–	C–H and O–H overtones	Advanced lipid degradation and rancidity detection	[37,38,39,40]

**Table 2:** Model statistics details of the variable selection/decorrelation procedure carried out by SELECT, corresponding to the optimal OLS regression model developed from NIR spectra after applying standard normal variate (SNV) as preprocessing technique. This model is proposed to quantify the carotenoids content of extra virgin olive oils, refined and virgin olive oils, sunflower oil, and high-oleic sunflower oil. (A) Chemometric details of the variable selection procedure. (B) Statistical characteristics of the developed model.

**(A) SELECT–OLS Modeling**			
Order of Selection	Predictor Index	Wavelength (nm)	Correlation Coefficient
1	403	1904	100.40
2	395	1888	−1469.66
3	558	2214	−114.92
4	531	2160	467.95
5	671	2440	97.09
6	561	2220	−1639.48
7	528	2154	727.28
8	527	2152	−1510.58
9	684	2466	−198.27
10	686	2470	654.11
11	61	1220	353.14
12	62	1222	−7859.98
13	57	1212	−3343.07
14	451	2000	−1010.39
15	303	1704	212.50
16	679	2456	220.25
17	677	2452	−424.75
18	575	2248	−206.62
19	647	2392	241.20
20	646	2390	−836.61
21	301	1700	−566.75
22	568	2234	1140.78
23	348	1794	−597.57
24	287	1672	1532.69
25	30	1158	−685.12
26	290	1678	−1977.23
27	524	2146	−1271.41
28	73	1244	−3531.89
29	534	2166	−2499.92
30	193	1484	1392.24
Intercept	9.80303		
**(B) Statistical Characteristics**			
Metric		Value	
Standard deviation of the error		1.54	
Mean absolute error		1.06	
Multiple correlation coefficient (R)		0.96	
Leave-one-out (LOO) validation		Value	
Residual standard deviation		1.73	
Mean prediction error		1.36	

## Data Availability

Data will be available upon reasonable request.

## Supplementary Materials

The following supporting information can be downloaded at: https://www.mdpi.com/article/10.3390/foods14132152/s1, Figure S1: NIR spectra of oil samples; Table S1: Chemometric details of the variable selection/decorrelation procedure carried out by SELECT, corresponding to the optimal OLS regression model developed from NIR spectra after applying standard normal variate (SNV) as preprocessing technique. This model is proposed to quantify the chlorophyll content of extra virgin olive oils, refined and virgin olive oils, sunflower oil, and high-oleic sunflower oil. (A) Chemometric details of the variable selection procedure. (B) Statistical characteristics of the developed model; Table S2: Chemometric details of the variable selection/decorrelation procedure carried out by SELECT, corresponding to the optimal OLS regression model developed from NIR spectra after applying standard normal variate (SNV) as preprocessing technique. This model is proposed to quantify the antioxidant activity content of extra virgin olive oils, refined and virgin olive oils, sunflower oil, and high-oleic sunflower oil. (A) Chemometric details of the variable selection procedure. (B) Statistical characteristics of the developed model; Table S3: Chemometric details of the variable selection/decorrelation procedure carried out by SELECT, corresponding to the optimal OLS regression model developed from NIR spectra after applying standard normal variate (SNV) as preprocessing technique. This model is proposed to quantify the rancidity sensorial attribute of extra virgin olive oils, refined and virgin olive oils, sunflower oil, and high-oleic sunflower oil. (A) Chemometric details of the variable selection procedure. (B) Statistical characteristics of the developed model; Table S4: Chemometric details of the variable selection/decorrelation procedure carried out by SELECT, corresponding to the optimal OLS regression model developed from NIR spectra after applying standard normal variate (SNV) as preprocessing technique. This model is proposed to quantify the fruity green of extra virgin olive oils, refined and virgin olive oils, sunflower oil, and high-oleic sunflower oil. (A) Chemometric details of the variable selection procedure. (B) Statistical characteristics of the developed model; Table S5: Chemometric details of the variable selection/decorrelation procedure carried out by SELECT, corresponding to the optimal OLS regression model developed from NIR spectra after applying standard normal variate (SNV) as preprocessing technique. This model is proposed to quantify the fruity ripe of extra virgin olive oils, refined and virgin olive oils, sunflower oil, and high-oleic sunflower oil. (A) Chemometric details of the variable selection procedure. (B) Statistical characteristics of the developed model; Table S6: Chemometric details of the variable selection/decorrelation procedure carried out by SELECT, corresponding to the optimal OLS regression model developed from NIR spectra after applying standard normal variate (SNV) as preprocessing technique. This model is proposed to quantify the bitter of extra virgin olive oils, refined and virgin olive oils, sunflower oil, and high-oleic sunflower oil. (A) Chemometric details of the variable selection procedure. (B) Statistical characteristics of the developed model; Table S7: Chemometric details of the variable selection/decorrelation procedure carried out by SELECT, corresponding to the optimal OLS regression model developed from NIR spectra after applying standard normal variate (SNV) as preprocessing technique. This model is proposed to quantify the pungency sensorial attribute of extra virgin olive oils, refined and virgin olive oils, sunflower oil, and high-oleic sunflower oil. (A) Chemometric details of the variable selection procedure. (B) Statistical characteristics of the developed model.
