# Supplementary material for: The Quality Prediction of Olive and Sunflower Oils Using NIR Spectroscopy and Chemometrics: A Sustainable Approach
Source: Foods. 2025 Jun 20;14(13):2152. doi: 10.3390/foods14132152 (PMC12248787; doi:10.3390/foods14132152)
Supplement: Supplementary file 1 [file foods-14-02152-s001.zip › foods-3675693-supplementary.pdf]

Supplementary data for:

Article

Quality Prediction of Olive and Sunflower Oils Using NIR Spectroscopy and Chemometrics: A Sustainable Approach

Taha Mehany, José M. González-Sáiz and Consuelo Pizarro *

Department of Chemistry, University of La Rioja, 26006 Logroño, Spain;
taha.abdellatif@unirioja.es, josemaria.gonzalez@unirioja.es
*Correspondence: consuelo.pizarro@unirioja.es; Tel.: +34 941299626

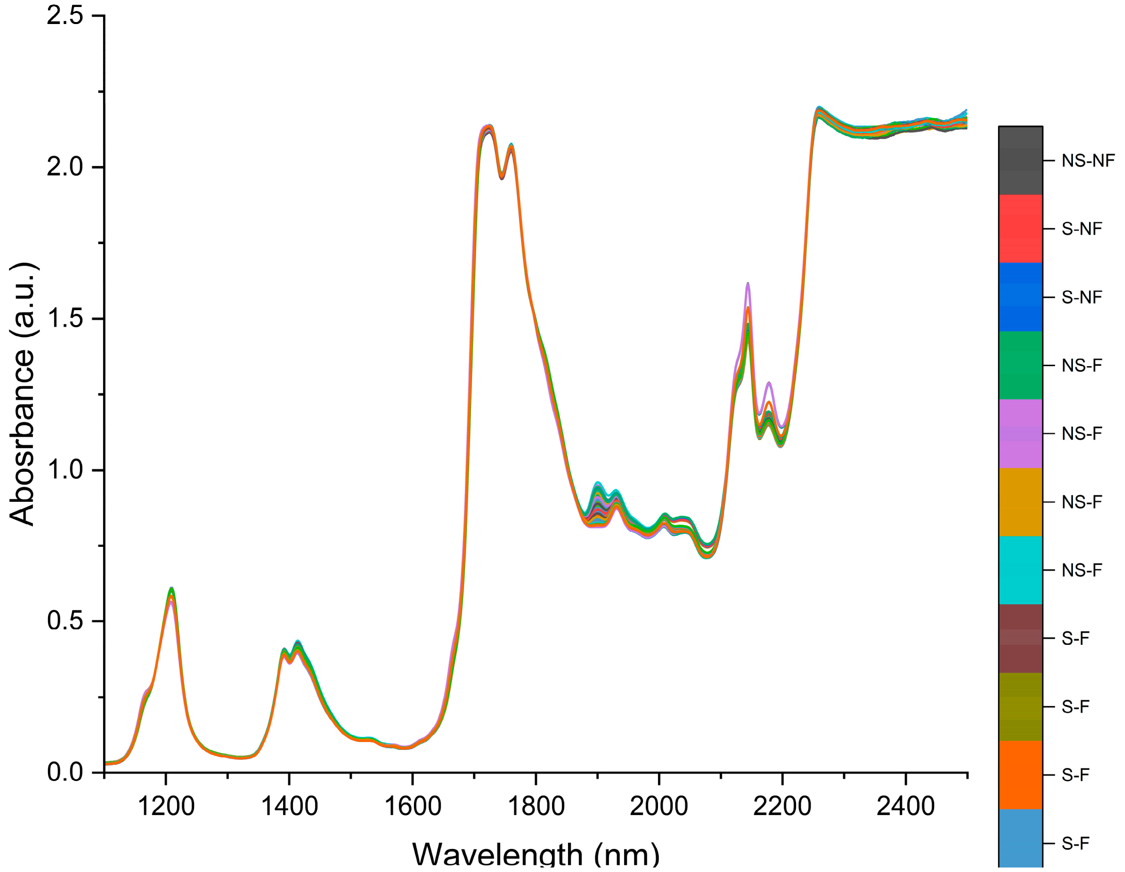

Figure S1. NIR spectra of oil samples. Where, NS-NF: Non-supplemented, non-fried; S-NF: Supplemented, non-fried; NS-F: Non-supplemented, fried; S-F: Supplemented, fried (this caption correspond to EVOO Picual.

Table S1. Chemometric details of the variable selection/decorrelation procedure carried out by SELECT, corresponding to the optimal OLS regression model developed from NIR spectra after applying standard normal variate (SNV) as preprocessing technique. This model is proposed to quantify the chlorophyll content of extra virgin olive oils, refined and virgin olive oils, sunflower oil, and high-oleic sunflower oil. (A) Chemometric details of the variable selection procedure. (B) Statistical characteristics of the developed model.

**(A) SELECT-OLS Modeling**			
**Order of Selection**	**Predictor Index (Spectral variable name)**	**Wavelength (nm)**	**Correlation Coefficient**
1	417	1932	541.49
2	419	1936	-17355.08
3	415	1928	-15905.62
4	418	1934	26297.24
5	138	1374	-1044.19
6	137	1372	27351.17
7	506	2110	-534.62
8	140	1378	8590.82
9	507	2112	3061.45
10	519	2136	-2548.93
11	187	1472	7594.96
12	39	1176	-7463.67
13	303	1704	597.15
14	493	2084	-1630.11
15	500	2098	6262.79
16	517	2132	-2963.85
17	497	2092	-23040.09
18	487	2072	5387.43
19	458	2014	-4449.08
20	451	2000	3589.64
21	181	1460	13928.08
22	421	1940	6669.29
23	446	1990	-14595.56
24	184	1466	-22297.69
25	145	1388	7031.72
26	264	1626	7090.78
27	259	1616	-29020.59
28	525	2148	1073.00
29	61	1220	4449.73
30	64	1226	-12361.25
Intercept	28.18457		
**(B) Statistical Characteristics**			

Metric	Value
Standard deviation of the error	4.31
Mean absolute error	3.03
Multiple correlation coefficient (R)	0.95
Leave one out (LOO) validation	Value
LOO Residual standard deviation	4.86
LOO Mean prediction error	3.88

Table S2. Chemometric details of the variable selection/decorrelation procedure carried out by SELECT, corresponding to the optimal OLS regression model developed from NIR spectra after applying standard normal variate (SNV) as preprocessing technique. This model is proposed to quantify the antioxidant activity content of extra virgin olive oils, refined and virgin olive oils, sunflower oil, and high-oleic sunflower oil. (**A**) Chemometric details of the variable selection procedure. (**B**) Statistical characteristics of the developed model.

**(A) SELECT-OLS Modeling**			
**Order of Selection**	**Predictor Index (Spectral variable name)**	**Wavelength (nm)**	**Correlation Coefficient**
1	148	1394	3214.27
2	158	1414	-1806.20
3	496	2090	3232.90
4	265	1628	-3055.01
5	378	1854	-4737.33
6	258	1614	30172.73
7	500	2098	8147.36
8	277	1652	-4546.83
9	293	1684	5814.39
10	499	2096	-43431.74
11	254	1606	-51484.99
12	525	2148	847.96
13	469	2036	-2305.63
14	467	2032	29850.35
15	257	1612	-52124.91
16	251	1600	44644.98
17	243	1584	-34256.87
18	242	1582	42018.08
19	464	2026	-17287.54
20	484	2066	13760.99
21	494	2086	-26716.19
22	465	2028	25398.47
23	679	2456	368.19
24	341	1780	-1182.47
25	345	1788	7609.80
26	478	2054	-23036.74
Intercept		69.84500	

**(B) Statistical Characteristics**	
**Metric**	**Value**
Standard deviation of the error	7.17
Mean absolute error	5.20
Multiple correlation coefficient (R)	0.96

Leave one out (LOO) validation	Value
LOO Residual standard deviation	8.00
LOO Mean prediction error	6.44

Table S3. Chemometric details of the variable selection/decorrelation procedure carried out by SELECT, corresponding to the optimal OLS regression model developed from NIR spectra after applying standard normal variate (SNV) as preprocessing technique. This model is proposed to quantify the rancidity sensorial attribute of extra virgin olive oils, refined and virgin olive oils, sunflower oil, and high-oleic sunflower oil. (A) Chemometric details of the variable selection procedure. (B) Statistical characteristics of the developed model.

**(A) SELECT-OLS Modeling**			
**Order of selection**	**Predictor Index (Spectral variable name)**	**Wavelength (nm)**	**Correlation Coefficient**
1	402	1902	-53.40
2	27	1152	-148.56
3	342	1782	107.54
4	344	1786	-1211.67
5	407	1912	168.77
6	191	1480	-589.17
7	396	1890	1337.14
8	557	2212	88.32
9	539	2176	-343.24
10	148	1394	-995.24
11	345	1788	1597.42
12	676	2450	-77.47
13	635	2368	119.01
14	339	1776	-788.27
15	509	2116	277.21
16	522	2142	-734.56
17	34	1166	2029.24
18	564	2226	-1079.06
19	531	2160	-654.59
20	529	2156	1388.70
21	406	1910	3345.60
22	347	1792	1346.18
23	659	2416	-83.49
24	408	1914	-2321.67
25	589	2276	95.59
26	604	2306	-457.98
27	150	1398	-2246.54
28	543	2184	640.87
29	665	2428	116.70
Intercept		4.26549	

**(B) Statistical Characteristics**	
Metric	Value

Standard deviation of the error	0.95
Mean absolute error	0.69
Multiple correlation coefficient (R)	0.95
Leave one out (LOO) validation	Value
LOO Residual standard deviation	1.07
LOO Mean prediction error	0.88

Table S4. Chemometric details of the variable selection/decorrelation procedure carried out by SELECT, corresponding to the optimal OLS regression model developed from NIR spectra after applying standard normal variate (SNV) as preprocessing technique. This model is proposed to quantify the fruity green of extra virgin olive oils, refined and virgin olive oils, sunflower oil, and high-oleic sunflower oil. (A) Chemometric details of the variable selection procedure. (B) Statistical characteristics of the developed model.

**(A) SELECT-OLS Modeling**			
**Order of Selection**	**Predictor Index (Spectral variable name)**	**Wavelength (nm)**	**Correlation Coefficient**
1	397	1892	29.99
2	405	1908	-232.28
3	655	2408	83.46
4	684	2466	-64.94
5	604	2306	114.60
6	619	2336	-620.56
7	650	2398	-189.86
8	319	1736	77.46
9	383	1864	-225.50
10	654	2406	-254.41
11	322	1742	-267.05
12	579	2256	108.79
13	181	1460	105.02
14	681	2460	158.99
15	615	2328	-316.27
16	659	2416	146.89
17	656	2410	-332.63
18	323	1744	-442.73
19	641	2380	-129.99
20	644	2386	189.07
21	407	1912	-708.34
22	585	2268	-249.59
23	586	2270	353.83
24	395	1888	2256.00
25	700	2498	68.83
26	180	1458	-2348.87
27	175	1448	678.45
28	178	1454	-4021.65
29	171	1440	-1308.69
30	645	2388	256.78
Intercept		1.13592	

**(B) Statistical Characteristics**	
Metric	Value
Standard deviation of the error	0.89
Mean absolute error	0.64
Multiple correlation coefficient (R)	0.88
Leave one out (LOO) validation	Value
LOO Residual standard deviation	1.01
LOO Mean prediction error	0.82

Table S5. Chemometric details of the variable selection/decorrelation procedure carried out by SELECT, corresponding to the optimal OLS regression model developed from NIR spectra after applying standard normal variate (SNV) as preprocessing technique. This model is proposed to quantify the fruity ripe of extra virgin olive oils, refined and virgin olive oils, sunflower oil, and high-oleic sunflower oil. (A) Chemometric details of the variable selection procedure. (B) Statistical characteristics of the developed model.

(A) SELECT-OLS Modeling

Order of Selection	Predictor Index (Spectral variable name)	Wavelength (nm)	Correlation Coefficient
1	419	1936	50.38
2	414	1926	-707.21
3	670	2438	50.99
4	220	1538	-159.20
5	219	1536	4962.39
6	194	1486	415.45
7	417	1932	969.37
8	663	2424	-47.11
9	347	1792	137.98
10	339	1776	-203.31
11	502	2102	-58.08
12	659	2416	148.84
13	45	1188	-451.29
14	191	1480	3986.04
15	420	1938	-2478.23
16	700	2498	-50.12
17	348	1794	489.40
18	351	1800	-336.29
19	356	1810	710.55
20	338	1774	764.88
21	231	1560	-2758.08
22	230	1558	5761.32
23	190	1478	3134.54
24	187	1472	-3953.78
25	188	1474	4734.99
26	326	1750	169.24
27	336	1770	-619.17
28	227	1552	4085.58
29	340	1778	536.76
30	306	1710	207.84
Intercept		0.92394	

(B) Statistical Characteristics

Metric	Value
Standard deviation of the error	0.79
Mean absolute error	0.56
Multiple correlation coefficient (R)	0.87
Leave one out (LOO) validation	Value
LOO Residual standard deviation	0.90
LOO Mean prediction error	0.72

Table S6. Chemometric details of the variable selection/decorrelation procedure carried out by SELECT, corresponding to the optimal OLS regression model developed from NIR spectra after applying standard normal variate (SNV) as preprocessing technique. This model is proposed to quantify the bitter of extra virgin olive oils, refined and virgin olive oils, sunflower oil, and high-oleic sunflower oil. (A) Chemometric details of the variable selection procedure. (B) Statistical characteristics of the developed model.

(A) SELECT-OLS Modeling

Order of Selection	Predictor Index	Wavelength (nm)	Correlation Coefficient
1	401	1900	23.68
2	559	2216	-32.23
3	395	1888	-426.52
4	558	2214	1235.27
5	65	1228	121.91
6	393	1884	-1118.60
7	146	1390	-1113.13
8	530	2158	80.48
9	267	1632	-592.85
10	398	1894	2313.09
11	241	1580	1106.61
12	268	1634	5916.45
13	237	1572	-4705.01
14	357	1812	-282.98
15	248	1594	-2542.75
16	264	1626	4686.29
17	247	1592	6606.06
18	565	2228	316.77
19	553	2204	-776.01
20	571	2240	-194.35
21	253	1604	3714.01
22	216	1530	1613.56
23	508	2114	-360.39
24	641	2380	-84.91
25	354	1806	585.47
26	68	1234	2691.97
27	234	1566	-3736.17
28	262	1622	-4168.24
29	255	1608	3909.96
30	643	2384	145.58
Intercept		1.48310	

(B) Statistical Characteristics

Metric	Value
Standard deviation of the error	0.87
Mean absolute error	0.57
Multiple correlation coefficient (R)	0.88
Leave one out (LOO) validation	Value
LOO Residual standard deviation	0.98
LOO Mean prediction error	0.73

Table S7. Chemometric details of the variable selection/decorrelation procedure carried out by SELECT, corresponding to the optimal OLS regression model developed from NIR spectra after applying standard normal variate (SNV) as preprocessing technique. This model is proposed to quantify the pungency sensorial attribute of extra virgin olive oils, refined and virgin olive oils, sunflower oil, and high-oleic sunflower oil. (A) Chemometric details of the variable selection procedure. (B) Statistical characteristics of the developed model.

**(A) SELECT-OLS Modeling**			
**Order of Selection**	**Predictor Index (Spectral variable name)**	**Wavelength (nm)**	**Correlation Coefficient**
1	505	2108	-97.83
2	573	2244	131.87
3	148	1394	92.53
4	155	1408	-379.40
5	143	1384	-409.05
6	380	1858	-364.93
7	308	1714	60.06
8	309	1716	-185.17
9	612	2322	50.76
10	637	2372	-108.37
11	312	1722	-395.97
12	596	2290	-305.58
13	226	1550	553.82
14	601	2300	283.03
15	600	2298	-457.96
16	634	2366	149.91
17	98	1294	-714.96
18	640	2378	231.17
19	100	1298	4921.73
20	213	1524	-2131.50
21	617	2332	313.01
22	214	1526	4988.76
23	641	2380	-207.18
24	627	2352	-102.44
25	685	2468	101.31
26	215	1528	-2731.87
27	595	2288	-228.43
28	212	1522	3022.52
29	89	1276	2027.66
30	85	1268	-3573.37
Intercept		1.76479	

**(B) Statistical Characteristics**	
Metric	Value
Standard deviation of the error	0.91
Mean absolute error	0.68
Multiple correlation coefficient (R)	0.86
Leave one out (LOO) validation	Value
LOO Residual standard deviation	1.08
LOO Mean prediction error	0.86
